# 

**Published:** 1935

**Authors:** 


### The Bristol Medico-Chirurgical Journal

A JOURNAL OF THE MEDICAL SCIENCES FOR THE
WEST OF ENGLAND AND SOUTH WALES.

PUBLISHED UNDER THE AUSPICES OF
*THE BRISTOL MEDICO-CHIRURGICAL SOCIETY.*

EDITOR:
J. A. NIXON, C.M.G., M.D., F.R.C.P.

WITH WHOM ARE ASSOCIATED

E. W. HEY GROVES, D.Sc., M.S., F.R.C.S.
A. E. ILES, O.B.E., M.B., B.S., F.R.C.S.
A. RENDLE SHORT, M.D., B.S., B.Sc., F.R.C.S.
E. WATSON-WILLIAMS, M.C., Ch.M., F.R.C.S., Assistant Editor.

EDITORIAL SECRETARY:
A. L. FLEMMING, M.B., Ch.B.

---

"Scire est nescire, nisi id me

Scire alius sciret."

---

*VOL. LII.*

BRISTOL: J. W. ARROWSMITH LTD.
London: J. W. Arrowsmith (London) Ltd.

### Contents of Vol. LII.

*Page*

The Problem of Man's Origin. By A. Rendle Short, M.D., F.R.C.S. 1

Licence to Practise and Liberty to Teach Medicine in the English Provinces. By J. A. Nixon, C.M.G., M.D., F.R.C.P. .. .. 19

Habit and Illness. By H. H. Carleton, M.D., M.R.C.P. .. .. 41

Men-Midwives of the Past. By Miles H. Phillips, M.D., F.R.C.S., F.C.O.G. .. .. .. .. .. .. .. .. .. 83

Eczema and Dermatitis. By Norman Burgess, M.D., M.R.C.P. .. 103

Medical Treatment of Hyperthyroidism. By J. Innes Noble, M.B., Ch.B., F.R.C.S. .. .. .. .. .. .. .. .. 113

The Evolution of British Orthopædics. By Sir Humphry Rolleston, Bart., G.C.V.O., K.C.B., M.D. .. .. .. .. .. 143

Diseases of the Lachrymal Sac. By A. E. Iles, O.B.E., M.B., F.R.C.S. 151

Treatment of Lachrymal Obstruction. By Gordon R. Scarff, M.B., Ch.B., F.R.C.S. .. .. .. .. .. .. .. .. 159

Thomas Marryat, M.D.: A Memoir. By A. S. MacNalty, M.D., F.R.C.P. .. .. .. .. .. .. .. .. .. 165

The Twenty-fourth Long Fox Memorial Lecture: Observations on Pain. By Macdonald Critchley, M.D., F.R.C.P. .. .. 192

Results of the Treatment of Mental Conditions. By G. de M. Rudolf, M.R.C.P., D.P.M., D.P.H. .. .. .. .. .. .. 219

Clinical Record: A Case of Pulmonary Consolidation of Doubtful Origin. By B. Hudson, M.D., M.R.C.P., and F. L. Wollaston, M.D. 233

Reviews of Books .. .. .. .. .. .. 57, 125, 171, 236

Editorial Notes .. .. .. .. .. .. 63, 130, 181, 244

Obituaries .. .. .. .. .. .. .. 65, 181, 248

Meetings of Societies .. .. .. .. .. 67, 134, 182, 251

The Medical Library of the University of Bristol 69, 135, 186, 255

Publications Received .. .. .. .. .. 72, 139, 188, 257

Local Medical Notes .. .. .. .. .. 73, 140, 189, 259

### The Medical Library of the University of Bristol,

WITH WHICH IS INCORPORATED

**The Library of the Bristol Medico-Chirurgical Society.**

---

*The following donations have been received since the publication of the list in December,* 1934.

March, 1935.

| | |
|---|---|
| American Laryngological, Rhinological and Otological Society, Inc. (1) .. .. .. | 1 volume. |
| American Otological Society (2) .. .. | 1 volume. |
| Association of American Physicians (3) .. | 1 volume. |
| Dr. W. S. Bainbridge (4) .. .. .. | 1 volume. |
| Professor R. J. Brocklehurst (5) .. .. | 17 volumes. |
| J. Michell Clarke Fund (6) .. .. .. | 1 volume. |
| Professor E. W. Hey Groves (7) .. .. | 1 volume. |
| M. Auguste Lumière (8) .. .. .. .. | 1 volume. |
| Mrs. J. E. MacKenty (9).. .. .. .. | 1 volume. |
| Professor C. Bruce Perry (10) .. .. .. | 2 volumes. |
| Dr. J. O. Symes (11) .. .. .. .. | 1 volume. |
| Dr. W. Roger Williams (12) .. .. .. | 1 volume. |

Unbound periodicals have also been received from Professor E. W. Hey Groves, Professor C. Bruce Perry and Dr. J. Odery Symes.

### THE ONE HUNDRED AND FIFTIETH LIST OF BOOKS.

The figures in round brackets refer to the figures after the names of the donors. The books to which no such figures are attached have either been bought from the Library Fund or received through the *Journal*.

| | | | |
|---|---|---|---|
| **Aarons, S. J.** .. .. .. | *Aids to Gynæcology* .. .. | 5th Ed. (5) | 1920 |
| **Adam, J.** .. .. .. | *Asthma and its Radical Treatment* .. .. | | 1913 |
| **Agduhr, E.** .. .. .. | *Zur Kenntnis des Einflusses der Graviditäten und des Ergosterins auf das Wachstum* .. | | 1932 |
| **Atkinson, E. M.** .. .. | *Abscess of the Brain* .. .. .. .. .. .. | | 1934 |
| **Bailey, H.** .. .. .. | *Demonstrations of Physical Signs in Clinical Surgery* .. .. .. .. .. .. | 4th Ed. | 1933 |
| **Barnett, H. N.** .. .. | *The Student's Text-book of Surgery* .. .. | | [1916] |
| **Beeston, G. N.** .. .. | *The Conjoint Finals,* 1911–1932 | 2nd Ed. | 1933 |
| **Berkart, J. B.** .. .. | *On Asthma* .. .. .. .. .. .. .. .. .. | | 1878 |

**Bernard, C.** .. .. .. *Introduction to the Study of Experimental Medicine* .. .. .. .. .. .. .. .. .. 1927

**Broomhead, R.** .. .. *A Summary of the Treatment of Fractures and Dislocations* .. .. .. .. .. .. [1934]

**Burroughs, Wellcome and Co.** *The Romance of Exploration and Emergency First-Aid* .. .. .. .. .. [1934]

**Cameron, A. T.** .. .. *Recent Advances in Endocrinology* .. (11) 1933

**Campbell, H.** .. .. *Aids to Pathology* .. .. 4th Ed. (5) 1920

**Commission on Medical Education.** (Reports I—III.) .. .. .. 1927–28

**Crawford, A. M.** .. .. *Materia Medica for Nurses* .. 3rd Ed. 1934

**Cross, H. H. U.** .. .. *Electro-Therapy and Ionic Medication* .. 1925

**Crowley, R. H.** .. .. *Hygiene of School Life.* Ed. by C. W. Hutt .. .. .. .. .. .. 2nd Ed. (5) [1916]

**Czermak, J. N.** .. .. *Gesammelte Schriften.* 1 Bd. 2 Abt. .. 1879

**Da Costa, J.** .. .. .. *Clinical Hematology* .. .. .. .. .. .. 1902

**Dally, J. F. H.** .. .. *High Blood Pressure* .. .. .. 3rd Ed. 1934

**Edinburgh Pathological Club.** *An Inquiry into the Medical Curriculum* 1919

**Fairbrother, R. W.** .. *Handbook of Filterable Viruses* .. .. 1934

**Faulkner, A. B.** .. .. *Letters to the Royal College of Physicians* .. 1829

**Garrod, A. E. et al.** .. *Diseases of Children.* Ed. H. Thursfield and D. Paterson .. .. .. 3rd Ed. 1934

**Geddes, P., and Thomson, J.A.** *The Evolution of Sex* .. Rev. Ed. (5) 1901

**Gies, W. J.** .. .. .. *Dental Education in the U.S. and Canada* 1926

**Gray, H.** .. .. .. .. *Anatomy.* Ed. Pick and Howden. 16th Ed. 1905

**Heath, C.** .. .. .. *Practical Anatomy.* Ed. J. E. Lane. 9th Ed. (5) 1902

**Hoffman, F. L.** .. .. *Army Anthropometry and Medical Rejection Statistics* .. .. .. .. .. .. .. [1918]

**Hoffman, F. L.** .. .. *More Facts and Fallacies of Compulsory Health Insurance* .. .. .. .. .. .. [1920]

**Hoffman, F. L.** .. .. *San Francisco Cancer Survey. Preliminary Reports, I—VI* .. .. .. .. .. [1923–30]

**Hoffman, F. L.** .. .. *Suicide Problems* .. .. .. .. .. .. .. [1928]

**Hopewell-Ash, E. L.** .. *Melancholia in Everyday Practice* .. .. 1934

**Hudson, B.** .. .. .. *Aids to Medicine* .. .. .. 3rd Ed. (5) 1921

**Imperial Social Hygiene Congress.** *Proceedings* .. .. .. .. .. .. 1927

**International Orthodontic Congress.** 2. (1931) .. .. .. .. .. .. 1933

**Irvine, K. N.** .. .. *The B.C.G. Vaccine* .. .. .. .. .. .. .. 1934

**Jones, E.** .. .. .. *Treatment of the Neuroses* .. .. .. (5) 1920

**Katz, L. et al.,** .. .. *Handbuch der Speziellen Chirurgie des Ohres.* 1 Bd. 2 Hälfte .. .. 2te Aufl. 1913

**Lake, N. C., and Marshall, C. J.** *Surgical Anatomy and Physiology* 1934

**Leduc, J.** .. .. .. *Indications . . . du Transport par Avion dans les Affections Chirurgicales* .. (7) 1934

**Lord, J. R.** .. .. .. *The Clinical Study of Mental Disorders* .. 1926

**Lumière, A.** .. .. .. *La Renaissance de la Médecine Humorale* (8) 1935

**MacKenty, J. A.** .. .. *Malignant Disease of the Larynx* .. (9) 1934

**M'Lachlan, J.** .. .. *Applied Anatomy.* Rev. by A. A. S. Skirving .. .. 2 vols. 4th Ed. (5) 1908

**Martin, F. H.** .. .. *Fifty Years of Medicine and Surgery* .. (10) 1934

*Mayne's Medical Vocabulary.* Rev. by Wagstaffe and Parker 7th Ed. (5) 1902

**Moor, C. G., and Partridge, W.** *Aids to Bacteriology* 3rd Ed. (5) 1916
**Morris, H.** .. .. .. .. *Human Anatomy* .. .. .. .. 5th Ed. (5) [1914]
**Murrell, W.** .. .. .. .. *What to do in Cases of Poisoning: by P. Hamill* .. .. .. .. .. ..14th Ed. 1934
**Nall, S.** .. .. .. .. .. *Aids to Obstetrics.* Rev. by C. J. N. Longridge .. .. .. .. .. 8th Ed. (5) 1921
**Norton, S.** .. .. .. .. [*Seven Alchemical Tracts.*] Ed. E. Deani 1630
**Oliver, H. G.** .. .. *The Etiology and Treatment of Spasmodic Bronchial Asthma* .. .. .. .. .. .. 1934
**Peking Union Medical College.** *Dedication Ceremonies and Medical Conference* .. .. .. .. .. .. .. .. .. 1922
**Raebiger, H.** .. .. .. .. *Das Meerschweinchen ; seine Zucht, Haltung u. Krankheiten* .. .. .. .. .. 2te Aufl. 1933
**Rawling, L. B.** .. .. *Head Injuries* .. .. .. .. .. .. .. .. .. 1934
**Robertson, W. G. A.** .. *Aids to Forensic Medicine and Toxicology* 9th Ed. (5) 1922
**Robertson, W. G. A.** .. *Aids to Public Health* .. .. .. (5) 1923
**Samson, E.** .. .. .. .. *Progressive Practice* .. .. .. .. .. .. [1931]
**Semon, H. C. G.** .. .. *An Atlas of the Commoner Skin Diseases* .. 1934
**Stephens, J. W. W., and Christophers, S. R.** *The Practical Study of Malaria* .. .. .. .. .. .. .. .. (5) 1904
**Symonds, W. S.** .. .. *Old Bones, or Notes for Young Naturalists* 2nd Ed. (5) 1864
**Treves, F.** .. .. .. .. *Surgical Applied Anatomy.* Rev. by C. C. Choyce .. .. .. .. .. .. 9th Ed. 1934
**Walker, G. E.** .. .. *Essays in Ophthalmology* .. .. .. (12) 1879
**Whitnall, S. E.** .. .. *The Study of Anatomy* .. 2nd Ed. (6) 1933

### TRANSACTIONS, REPORTS, JOURNALS, ETC.

American Laryngological, Rhinological and Otological Society. Transactions of the 40th Annual Meeting .. .. .. .. (1) 1934
American Otological Society. Transactions. Vol. XXIV .. (2) 1934
L'Année Médicale Pratique, 1932 .. .. .. .. .. .. .. .. .. (10) 1932
Association of American Physicians. Transactions. Vol. 49 .. (3) 1934
Chinese Medical Directory. 3rd Edition .. .. .. .. .. .. .. .. 1932
Dentists' Register, 1921, 1922, 1924, 1930–1932 .. .. .. .. 1921–1932
Imperial Cancer Research Fund. Scientific Report, 11 and Supplement 1934
Kelly's Directory of Bristol, 1935 .. .. .. .. .. .. .. .. .. .. .. 1935
Liverpool Medical and Surgical Reports. Vol. I .. .. .. .. .. 1867
Medical Register, 1922, 1924, 1932 .. .. .. .. .. .. .. 1922–1932
Middlesex Hospital Medical School. Published Papers, 1927/8, 1928/9 1928–1929
Ophthalmological Society of the United Kingdom. Transactions. Vol. LIV .. .. .. .. .. .. .. .. .. .. .. .. .. .. .. .. .. 1934
Report on the 7th International Congress of Military Medicine, 1933 (4) 1934
Rockefeller Foundation Annual Report, 1920 .. .. .. .. .. .. 1920
Royal College of Physicians of London. List of Fellows, etc., 1920, 1921 1920–21
St. Bartholomew's Hospital Reports. Vol. LXVII .. .. .. .. .. 1934
Surgical Clinics of North America. Vol. 14. No. 6 (December, 1934) 1934

### Publications Received

From Messrs. Burroughs, Wellcome & Co.:

*The Romance of Exploration and Emergency First Aid.*

From Messrs. Cassell & Co. Ltd.:

*A Text-book of Gynæcological Surgery.* 3rd Edition. By C. Berkeley, Kt., M.A., M.D., and V. Bonney, M.S., M.D., D.Sc. Price 45s.

*Clinical Methods.* 10th Edition. By R. Hutchison, M.D., and D. Hunter, M.D. Price 12s. 6d.

From Messrs. Jowett & Sowry Ltd.:

*Summary of the Treatment of Fractures and Dislocations.* By R. Broomhead, M.B. Price 3s. 6d.

From Messrs. H. K. Lewis & Co. Ltd.:

*A Short Practice of Surgery.* 2nd Edition (in one volume). By H. Bailey and R. J. McN. Love, M.S. Price 30s.

*Injuries and Their Treatment.* By W. E. Tucker, M.A., B.Ch. Price 9s.

*Some Thoughts of a Doctor.* By F. P. Weber, M.A., M.D. Price 6s.

From Messrs. Macmillan & Co. Ltd.:

*The Conduct and Fate of the Peripheral Segment of a Divided Nerve in the Cervical Region when United by Suture to the Central Segment of Another Divided Nerve.* By C. Ballance, Kt. Price 7s. 6d.

From The Oxford University Press:

*X-Ray Interpretation.* By H. C. H. Bull, M.A., M.B. Price 21s.

*Oxygen and Carbon-Dioxide Therapy.* By A. Campbell, M.D., D.Sc., and E. P. Poulton, M.A., D.M. Price 12s. 6d.

*A Practical Manual of Diseases of the Chest.* By M. Davidson, M.A., M.D. Price 42s.

*Hugh Owen Thomas.* By F. Watson. Price 12s. 6d.

From Dr. M. Siegel:

*Constructive Eugenics and Practical Marriage.* By M. Siegel, M.D.

From Messrs. John Wright & Sons Ltd.:

*Synopsis of Obstetrics and Gynæcology.* 6th Edition. By A. W. Bourne, M.A., M.B., B.Ch. Price 15s.

*British Journal of Surgery.* Vol. XXII. No. 87. January, 1935. Price 12s. 6d.

*Ideal Health.* 3rd Edition. By A. Bryce, M.D., C.M. Price 5s.

*Biological Politics.* By F. W. Inman, M.B., Ch.B. Price 7s. 6d.

*The Status of Enzymes and Hormones in Therapy.* By G. E. Walker, M.D. Price 2s.

### Local Medical Notes

EXAMINATION RESULTS.

**University of Bristol.**—Students of the University have recently passed the following examinations :—

M.B., Ch.B. (*Part I.*).—*Second Examination. Pass :* Catherine M. Addy, Ruth Appleby, Dorothy M. Ayre, D. L. Bayley, Jean A. Butt, Mary Crago, J. N. P. Davies, Marjorie O. Dunster, J. L. Elliott, J. L. Emery, Sara M. Field-Richards, Joan M. Foss, E. M. Grace, Betty F. Hannaford, F. R. Hurford, Megan E. Jones, Rosemary W. Knowles, N. E. Melling, Joyce S. Miller, C. A. St. Hill, Jeannette D. Shed, Dorothy M. Shotton, S. J. Silvey, P. R. H. Slade, J. E. Smith, Sylvia E. Smith, A. A. Steinberg, Edith M. Wagstaff, T. H. White.

M.B., Ch.B. (*Part II.*).—*Second Examination. Pass :* J. F. Ackroyd, E. M. Barber, P. A. Bennett, Kathleen E. Byrt, M. Dworkis, W. H. G. Elliott, Eileen M. D. Knox, Jane Mackintosh, Joan E. Mackworth (with Distinction in Anatomy), G. R. E. Maxted, R. H. Owen, A. H. Wright (with Distinction in Anatomy).

B.D.S.—*Second Examination. Pass :* D. R. Stevens.

L.D.S.—*First Professional Examination. Pass :* F. S. S. Baguley, D. C. Bodenham, J. F. Durie, C. F. Howell, R. J. Lee, C. C. Moore, A. J. H. Orchard, J. C. F. Rolls, D. M. Sanders, J. G. Windmill.

**Conjoint Board.**—M.R.C.S., L.R.C.P.—*Pass : In Chemistry and Physics :* Susie C. Sampson. *In Chemistry :* Mary R. More. *In Physics and Biology :* S. G. Sheffield. *In Anatomy and Physiology :* T. R. de V. Vallentin, P. W. J. Parkes. *In Anatomy :* A. L. T. Beddoe, Margaret E. M. Slater. *In Physiology :* Myrtle M. Baxter. *In Pharmacology and Materia Medica :* P. W. M. Martin. *In Pathology and Surgery :* A. W. Woolley. *In Surgery :* A. E. Jowett. *In Pathology and Midwifery :* Kathleen G. Brimelow, J. G. Field. *In Midwifery :* T. H. Taylor.

L.D.S., R.C.S.—*Pass: First Professional Examination:* A. N. Brimelow, J. R. P. Thomas, J. T. S. Hutchins, W. White. *Final Examination* (*Part I.*)*:* D. J. Dymond, R. Edwards, J. G. Jones. *Final Examination* (*Part II.*)*:* L. Jonathan.

L.S.A.—*Final Examination. Pass: In Medicine:* R. M. Outfin.*

APPOINTMENTS.

**Bristol Royal Infirmary.**—Physician: Dr. A. T. Todd, O.B.E., M.R.C.P. Surgeon: Mr. A. W. Adams, M.S., F.R.C.S. Obstetric Physician: Mr. H. L. Shepherd, Ch.M. Assistant Physician: Dr. G. E. F. Sutton, M.C., M.D., M.R.C.P. Assistant Surgeon: Mr. R. G. Paul, F.R.C.S.

**Bristol General Hospital.**—Physician: Dr. J. A. Birrell, M.D., M.R.C.P. Assistant Physician: Dr. H. J. Orr-Ewing, M.C., M.D., F.R.C.P.

* Qualified.

### Bristol Medico-Chirurgical Society.

### President.

A. R. SHORT, B.Sc., M.D.Lond., F.R.C.S.

### President-Elect.

A. E. ILES, *O.B.E.*, M.B., B.S., F.R.C.S.

### Committee.

*Ex-Officio*
- J. A. NIXON, *C.M.G.*, B.A., M.D. Cantab., F.R.C.P. (Editor of *Journal*).
- G. PARKER, M.A., M.D. Cantab. (Hon. Librarian).

*Elected.*

H. ELWIN HARRIS, B.A., M.B. Cantab., F.R.C.S. (Ex-President).
DUNCAN WOOD, F.R.C.S.
A. L. FLEMMING, M.B., Ch.B. Bristol.
CLIFFORD A. MOORE, M.B., M.S. Lond.
H. L. FULLER.
P. S. MARTIN, *M.C.*
W. V. WOOD, *M.C.*, B.A.Cantab.
A. FELLS, M.B., C.M. Edin.
R. C. CLARKE, *O.B.E.*, M.B., Ch.B. Bristol.
A. L. TAYLOR, M.D. Lond.

### Honorary Secretary.

H. L. SHEPHERD, M.B., Ch.M. Brist.

### Honorary Treasurer.

A. E. ILES, *O.B.E.*, M.B., B.S. Lond., F.R.C.S.

### University Library Sub-Committee.

G. PARKER, M.A., M.D. Cantab.
A. R. SHORT, B.Sc., M.D. Lond., F.R.C.S.
J. O. SYMES, M.D. Lond.

Medical Practitioners wishing to join the Society are requested to communicate with the Hon. Secretary, Mr. H. L. SHEPHERD, 79 Pembroke Road, Clifton. The Annual Subscription is £1 11s. 6d., payable to Hon. Treasurer.

---

*Extracts from Journal By-laws.*

4.—The *Journal* shall be delivered free to Subscribers and also to Members of the Society whose subscriptions are not in arrear.

6.—The Annual Subscription to the *Journal* is 10/6, payable to Hon. Treasurer.

---

Communications intended for insertion in the *Journal* should be sent to the Editor, Dr. J. A. NIXON, 7 Lansdown Place, Clifton, Bristol.

Books for Review and Exchange Journals should be sent to the Assistant-Editor, *Bristol Medico-Chirurgical Journal*, Medical Library, The University, Bristol.

Communications referring to the delivery of the *Journal* should be sent to the Editorial Secretary, A. L. Flemming, 48 Pembroke Road, Clifton, Bristol.

Cases for Binding Vols. I—LI are now ready, and may be obtained price 1/9 each (postage extra), upon application to J. W. ARROWSMITH LTD., Quay Street, Bristol; or the *Journal* will be bound, including Case, for 7/- per volume.

### Bristol Medico-Chirurgical Society.

### PAST PRESIDENTS.

1874—75. FREDERICK BRITTAN, M.D., B.S. Dub.

1875—76. ROBERT WILLIAM COE.

1876—77. SAMUEL MARTYN, M.D. Ed.

1877—78. AUGUSTIN PRICHARD, M.D. Berlin.

1878—79. HENRY EDWARD FRIPP, M.D. Würzburg.

1879—80. WILLIAM MICHELL CLARKE.

1880—81. JOSEPH GRIFFITHS SWAYNE, M.D. Lond.

1881—82. EDWARD LONG FOX, M.D. Oxon.

1882—83. JAMES GEORGE DAVEY, M.D. St. And.

1883—84. WILLIAM JOHNSTONE FYFFE, M.D., B.A. Dub.

1884—85. GEORGE FORSTER BURDER, M.D. Aberd.

1885—86. WILLIAM HENRY SPENCER, M.D., M.A. Cantab.

1886—87. FRANCIS POOLE LANSDOWN.

1887—88. LEMUEL MATTHEWS GRIFFITHS.

1888—89. ROBERT SHINGLETON SMITH, M.D., B.Sc. Lond.

1889—90. NELSON CONGREVE DOBSON, Ch.M. Brist.

1890—91. SAMUEL HENRY SWAYNE.

1891—92. FRANCIS RICHARDSON CROSS, M.B. Lond., LL.D. Brist.

1892—93. EDWARD MARKHAM SKERRITT, M.D., B.S., B.A. Lond.

1893—94. JAMES GREIG SMITH, M.B., C.M., M.A. Aberd., F.R.S.E.

1894—95. ALFRED JAMES HARRISON, M.B. Lond.

1895—96. ARTHUR WILLIAM PRICHARD.

1896—97. ALFRED EDWARD AUST LAWRENCE, M.D., C.M. Aberd.

1897—98. JOHN EDWARD SHAW, M.B., C.M. Ed.

1898—99. ROBERT ROXBURGH, M.B. Ed.

1899—00. WILLIAM HENRY HARSANT.

1900—01. DAVID SAMUEL DAVIES, M.D. Lond.

1901—02. BARCLAY JOSIAH BARON, M.B., C.M. Ed.

1902—03. GEORGE MUNRO SMITH, M.D. Brist.

1903—04. JAMES PAUL BUSH, C.M.G., C.B.E., Ch.M. Brist.

1904—05. REGINALD EAGER, M.D. Lond.

1905—06. JOHN DACRE.

1906—07. JAMES TAYLOR.

1907—08. HENRY WALDO, M.D., C.M. Aberd.

1908—09. JOHN MICHELL CLARKE, M.D., M.A. Cantab.

1909—10. JAMES SWAIN, C.B., C.B.E., M.D., M.S. Lond.

1910—11. BERTRAM MITFORD HERON ROGERS, M.D., B.Ch., B.A. Oxon.

1911—12. CHARLES ALEXANDER MORTON.

1912—13. WALTER CARLESS SWAYNE, M.D., B.S. Lond. and Brist.

1913—14. PATRICK WATSON-WILLIAMS, M.D. Lond.

1914—15. WILLIAM HARRY CHRISTOPHER NEWNHAM, M.A., M.B. Cantab.

1915—16. GEORGE PARKER, M.A., M.D. Cantab.

1916—17. FRANCIS HENRY EDGEWORTH, M.A., M.D. Cantab, D.Sc. Lond.

1917—18. FREDERICK LACE.

1918—19. ROBERT GUTHRIE POOLE LANSDOWN, M.D., B.S. Durh.

1919—20. I. WALKER HALL, M.D., Ch.B. Vict.

1920—21. LEOPOLD ERNEST VALENTINE EVERY-CLAYTON, M.D. Lond.

1921—22. CYRIL HUTCHINSON WALKER, B.A., M.B. Cantab.

1922—23. JOHN ALEXANDER NIXON, C.M.G., B.A., M.D. Cantab.

1923—24. JOHN LACY FIRTH, M.D., M.S. Lond.

1924—25. JOHN ODERY SYMES, M.D. Lond.

1925—26. THOMAS CARWARDINE, M.S. Lond.

1926—27. ARTHUR LAUNCELOT FLEMMING, M.B., Ch.B. Brist.

1927—28. WALTER KENNETH WILLS, M.A., M.B., B.Ch. Cantab.

1928—29. HENRY LAWRENCE ORMEROD, M.D., B.Ch. R.U.I.

1929—30. CHARLES FERRIER WALTERS.

1930—31. DAVID CHARLES RAYNER, Ch.M. Brist.

1931—32. JOHN ROGER CHARLES, M.A., M.D. Cantab.

1932—33. ERNEST WILLIAM HEY GROVES, M.D., M.S., D.Sc., B.Sc. Lond.

1933—34. HERBERT ELWIN HARRIS, B.A., M.B. Cantab.

1935.

### LIST OF SUBSCRIBERS

TO THE

### Bristol Medico-Chirurgical Journal.

### HONORARY MEMBERS OF THE SOCIETY.

| | |
|---|---|
| *Swain, James, *C.B.*, *C.B.E.*, M.D., M.S. Lond. .. .. .. .. | Wyndham House, Easton-in-Gordano. |
| *Watson-Williams, P., M.D.Lond. | 2 Rodney Place, Clifton, Bristol 8. |

### SUBSCRIBERS.

*Those marked * are Members of the Society.*

| | |
|---|---|
| *Ackland, W. R., M.D.S. ... ... ... ... ... | 5 Rodney Place, Clifton, Bristol 8. |
| *Adams, A. W., M.S. Lond. ... ... ... ... ... | Rodney House, Clifton, Bristol 8. |
| Adams, J. B .... ... ... ... ... ... ... ... ... | Kent Lodge, Eastbourne. |
| *Adams, S. B., Ph.D. M.B., Ch.B. Brist. ... ... | 19 Charlotte Street, Bristol 1. |
| *Aldwinckle, H. M., M.B., Ch.B. Brist. ... | 1 Manor Road, Fishponds, Bristol. |
| *Alexander, A. J. P., M.D. Belf, ... ... ... | Winsley Sanatorium, near Bath. |
| *Alexander, D. A., M.B., Ch.B. Vict. ... ... | 112 Pembroke Road, Clifton, Bristol 8. |
| Alexander, G. L., B.A., M.B., B.Ch. Cantab. | West African Medical Service, Laura, via Accra, Gold Coast. |
| *Alford, A. F., M.B., Ch.B. Brist. ... ... ... | 14 Clifton Park, Bristol 8. |
| Alford, H. J., M.D. Lond.... ... ... ... ... | 4 Park Terrace, Taunton. |
| *Armstrong, Jean, M.B., Ch.B. Brist. ... ... | Succoth, Duckmoor Road, Ashton Gate, Bristol 3. |
| Astley-Weston, B.A., M.B., Ch.B.Brist. ... | High Meadow, Radbrook, Shrewsbury. |
| *Atkinson, E. Miles, F.R.C.S. Eng., M.B., B.S.Lond. ... ... ... ... ... ... ... ... ... | 9 Royal Crescent, Bath. |
| *Aubrey, T., M.B. Lond. ... ... ... ... ... ... | Bitton, near Bristol. |
| *Baker, Lily A., M.D., B.Ch., B.A.O., R.U.I. | Clifton Down House, Beaufort Buildings, Clifton, Bristol 8. |
| *Barber, M. C., M.B., Ch.B. Brist. ... ... ... | Ingledene, High Street, Staple Hill, Bristol. |
| *Barker, G. H., M.D., B.Sc. Lond. ... ... ... | 124 Redland Road, Bristol 6. |
| *Bartholomew, E. U. ... ... ... ... ... ... ... | 12 Lansdown Place, Clifton, Bristol 8. |
| *Bates, R. M.... ... ... ... ... ... ... ... ... | Purdown House, Stapleton, Bristol 5. |
| *Beattie, Myra K., M.D., B.Sc.Belf . . ... ... | 10 Cambridge Park, Redland, Bristol 6. |
| Beatson, D. H., M.B., Ch.B. Brist. ... ... ... | 8 Richmond Park Road, Clifton, Bristol 8. |
| *Bergin, F. G. ... ... ... ... ... ... ... ... | 31 Oakfield Road, Clifton, Bristol 8. |
| Bernard, C. ... ... ... ... ... ... ... ... ... | 564 Fishponds Road, Fishponds, Bristol. |
| Berry, F. C., M.D., B.Ch., B.A. Dub. ... ... | Locarno, Burnham, Somerset. |
| *Berry, R. J. A., Prof., M.D., C.M. ... ... ... | Director of Medical Services, Stoke Park Colony, Bristol 5. |
| *Birrell, J. A., M.D., M.S. Lond. ... ... ... | 36 Kellaway Avenue, Bristol 6. |
| *Blachford, J. V., *C.B.E.*, M.D. Durh. ... ... | Milverton House, Long Ashton, near Bristol. |
| Blackett, J. F., M.D., B.S. Lond. ... ... ... | Hamsterley, Bloomfield Park, Bath. |
| Bletchly, G. Playne, M.B. Lond. ... ... ... | Hazelwood, Nailsworth, Glos. |
| *Bodman, A. P., M.B., Ch.B. ... ... ... ... ... | 11 Hurle Crescent, Clifton, Bristol 8. |
| *Bodman, C. O., M.D. Durh. ... ... ... ... | 17 Upper Belgrave Road, Clifton, Bristol 8. |
| *Bodman, F. H., M.B., Ch.B. Brist. ... ... ... | 2 Redland Court Road, Bristol 6. |
| *Bodman, J. Hervey, M.D. Lond. ... ... ... | 11 Hurle Crescent, Clifton, Bristol 8. |
| Bolt, R. F. ... ... ... ... ... ... ... ... ... ... | 70 Great Russell Street, London, W.C.1. |
| *Boulton, B. J., M.B., Ch.B. Brist. ... ... ... | Ham Green Hospital, Pill, near Bristol. |
| *Bown, N. J., M.B., Ch.B. Brist.... ... ... ... | Deméter, Wraxall, Somerset. |
| *Bradbeer, E. G., M.B., Ch.B. Brist. ... ... | 13 Percival Road, Bristol 8. |
| *Bradley, W. H., B.A., D.M. Oxon. ... ... | St. Wulstan's, Stratton-on-Fosse, Bath. |

Brasher, C. W. J. ... ... ... ... ... ... Woodlands Park, Great Missenden, Bucks.
*Brinckman, W. P., M.B., Ch.B. Brist. ... ... 4 Oakland Road, Redland, Bristol 6.
*Britton, R. B., M.B., B.S. Lond. ... ... ... 33a Whiteladies Road, Clifton, Bristol 8.
*Brocklehurst, R. J., M.A., D.M. Oxon.... ... The University, Bristol 8.
Brown, J. E., M.B., Ch.B. Brist. ... ... ... 137 Belmont Circ Road, Port of Spain, Trinidad, B.W.I.
*Buckmaster, G. A., M.A., M.D. Oxon. ... ... 6 Victoria Square, Clifton, Bristol 8.
*Burges, R. ... ... ... ... ... ... ... ... ... Druid's Mead, Stoke Bishop, Bristol 9.
*Burgess, N. F. C., M.D. Cantab.... ... ... ... Hereford House, Clifton, Bristol 8.
*Burnett, R. H., M.B., B.S. Durh. ... ... ... 528 Bath Road, Brislington, Bristol 4.
*Bush, G. B., M.B., Ch.B. Brist. ... ... ... 132 Queen's Road, Clifton, Bristol 8.

*Cairns, F. J. ... ... ... ... ... ... ... ... ... Devon House, Whitehall Road, Bristol 5.
*Carey, R. S., *O.B.E.*, B.A. Cantab. ... ... 502 Bath Road, Brislington, Bristol 4.
*Carleton, H. H., M.A., M.D., B.Ch. Oxon. ... 1 Lansdown Road, Clifton, Bristol 8.
*Carwardine, Thomas, M.B., M.S. Lond.... ... 16 Victoria Square, Clifton, Bristol 8.
*Casson, E., M D., Ch. B. Brist. ... ... ... ... Dorset House, Clifton Down, Bristol 8.
*Cates, H. J., M.D. Lond. ... ... ... ... ... Northwoods House, Winterbourne, Bristol.
*Chambers, E. R. ... ... ... ... ... ... ... ... 9 Clifton Park, Clifton, Bristol 8.
*Charles, J. R., M.A., M.D. Cantab.... ... ... 5 Rodney Place, Clifton, Bristol 8.
*Chesterman, J. T., M.D., B.S. ... ... ... ... Glen Avon, Broomfield Road, Bath.
*Chitty, H., M.S. Lond. ... ... ... ... ... ... 46 Pembroke Road, Clifton, Bristol 8.
*Christofferson, P. E., M.B., Ch.B. Brist. ... 8 Lansdown Place, Clifton, Bristol 8.
*Clarke, R. C., *O.B.E.*, M.B., Ch.B. Brist. ... 29 Victoria Square, Clifton, Bristol 8.
Connellan, P. S. ... ... ... ... ... ... ... ... Marmion, Shortheath Road, Farnham, Surrey.
*Cooke, Elizabeth M., M.D. ... ... ... ... Failand Lodge, Guthrie Road, Clifton.
*Cooke, R. V., Ch.M. ... ... ... ... ... ... ... Failand Lodge, Guthrie Road, Clifton.
*Cookson, R. G. ... ... ... ... ... ... ... ... 5 Worcester Road, Clifton, Bristol 8.
*Coombs, N. C. ... ... ... ... ... ... ... ... 14 Southfield Road, Westbury-on-Trym, Bristol.
*Cooper, William Russell ... ... ... ... ... 13 Westfield Park, Redland, Bristol 6.
*Corfield, C., M.D. Durh. ... ... ... ... ... 5 Kensington Place, Clifton, Bristol 8.
*Cossham, W. L., M.B., Ch.B. Brist. ... ... 3 Claremont Road, Bishopston, Bristol 7.
*Courtney, R. M., M.B., Ch.B. Glasg. ... ... 487 Gloucester Road, Bristol 7.
*Coxon, Beatrice .. ... ... ... ... ... ... ... 16 Richmond Hill, Clifton, Bristol 8.
*Crook, B. A., M.B., Ch.B.Brist. ... ... ... Timsbury.
*Crossman, F. W., M.B. Durh. ... ... ... ... White's Hill, Hambrook, near Bristol.
*Cross, Clara D., M.D. ... ... ... ... ... ... 17 Midford Road, Odd Down, Bath.

*Dacre, John ... ... ... ... ... ... ... ... ... 14 Eaton Crescent, Clifton, Bristol 8.
*Datta, S. S. M.D. Brist. ... ... ... ... ... Snowdon House, Fishponds, Bristol.
*Davies, E. D. D. ... ... ... ... ... ... ... ... Standish House, Stonehouse, Glos.
*Dawson, W. R., *O.B.E.*, M.D. Dub.... ... ... 18 Brock Street, Bath.
Devine, H., *O.B.E.*, M.D. Lond. ... ... ... Holloway Sanatorium, Virginia Water.
*Dick, A., M.B., Ch.B. Glasg. ... ... ... ... 14 Owen Grove, Henleaze, Bristol.
*Dickson, W. A., M.D. ... ... ... ... ... ... Shire Hall, Gloucester.
*Dix, C. ... ... ... ... ... ... ... ... ... ... 11 Victoria Square, Clifton, Bristol 8.
*Dixon, Helen M., M.B., Ch.B. Brist. ... ... 10 Whatley Road, Clifton, Bristol 8.
*Dixon, T. B. ... ... ... ... ... ... .. ... 11 Pembroke Road, Clifton, Bristol 8.
*Drew, J. H., M.D. ... ... ... ... ... ... Penlee, Uphill Road, Weston-super-Mare.
*Duerden, J. R., M.B., Ch.B. Brist. ... ... 149 Bell Hill Road, St. George, Brisiol 5.
*Dutton, Hilda R. ... ... ... ... ... ... ... 405 Stapleton Road, Brlstol 5.
*Dyer, Walter Percy... ... ... ... ... ... ... West View, Westbury-on-Trym, Bristol.

*Eglinton, J. H. C. ... ... ... ... ... ... ... Street, Somerset.
Elliot Bros. Ltd. ... ... ... ... ... ... ... 20–22 O'Connell Street, Sydney, N.S.W., Australia.
Elliott, F. Percy, M.B., C.M. Aberd. ... ... 113 Grove Rd., Walthamstow, London, E.17.
*Elliott, W. H. A., M.B., B.S. Lond.... ... ... 116 Pembroke Road, Cilfton, Bristol 8.

| | |
|---|---|
| *Fallon, Col. J. ... ... ... ... ... ... ... ... | 35 Henleaze Gardens, Henleaze, Bristol. |
| *Faraker, E. C. ... ... ... ... ... ... ... ... | 85 Coldharbour Road, Westbury Park Bristol 6. |
| Farnfield, W. W. ... ... ... ... ... ... ... | Clive Vale, Gillingham, Dorset. |
| *Farquharson, J. L. ... ... ... ... ... ... ... | Breda, Weston-super-Mare. |
| *Fawn, G. F. ... ... ... ... ... ... ... ... ... | 6 Oakfield Road, Clifton, Bristol 8. |
| *Fayle, B. W. D., M.B., Ch.B. ... ... ... ... | Reine Barnes, Dursley, Glos. |
| *Fells, A., M.B., C.M. Edin. ... ... ... ... | 6 Elton Road, Clifton, Bristol 8 |
| *Fells, G. R., M.B., Ch.B. Brist. ... ... ... | 19 Southmead Road, Bristol. |
| *Fells, R. R., M.B., B.S. Lond. ... ... ... | 17 Mortimer Road, Clifton, Bristol 8 |
| *Feneley, G. L., M.B., Ch.B. ... ... ... ... | Englewood, Falcondale Road, Westbury-on-Trym. |
| *Firth, J. L., M.D., M.S. Lond. ... ... ... | 8 Victoria Square, Clifton, Bristol 8. |
| *Fisher, A. C., M.B., Ch.B. Brist. ... ... ... | Roan Antelope Mine, N. Rhodesia. |
| *FitzGibbon, G. M., M.D. ... ... ... ... ... ... | 52 Pembroke Road, Clifton, Bristol 8. |
| *Fitzgibbon, Mrs. L. P., M.B., Ch.B. ... ... | 52 Pembroke Road, Clifton, Bristol 8. |
| *Flemming, A. A. G. ... ... ... ... ... ... ... | Manver's House, Bradford-on-Avon. |
| *Flemming, A. L., M.B., Ch.B. Brist. ... ... | 48 Pembroke Road, Clifton, Bristol 8. |
| *Flemming, C. E. S. ... ... ... ... ... ... ... | Manver's House, Bradford-on-Avon. |
| *Forrester-Brown, Maud, M.D., M.S. Lond. ... | 22 Coombe Park, Bath. |
| *Foss, G. L., M.B., B.Ch. Camb. ... ... ... ... | Cloud's Hill House, St. George, Bristol 5. |
| *Fox, F. E., B.A. Camb. ... ... ... ... ... ... | Brislington House, Brislington, Bristol 4. |
| *Fraser, A. D., M.A., M.B., Ch.B. Glasg. ... | 4 Alexandra Road, Clifton, Bristol 8. |
| *Fraser, D., M.B., Ch.B. Edin. ... ... ... ... | Westgate, Dursley, Glos. |
| *Freeth, H., M.D. Dub. ... ... ... ... ... | 93 Redland Road, Bristol 6. |
| *Fuller, H. L. ... ... ... ... ... ... ... ... | 9 Gay Street, Bath. |
| *Furnival, Col. C. H. ... ... ... ... ... ... ... | "Yatton," Wellington Hill, Horfield, Bristol 7. |
| *Garden, R. R., M.A., M.B., B.Ch. Aberd. ... | 9 Harley Place, Clifton, Bristol 8. |
| Garland, E. B., M.B., C.M. Edin. ... ... ... | 114a Hampton Road, Redland, Bristol 6. |
| *Gee, C. A. H., M.B., Ch.B. Brist. ... ... ... | Wansdyke, Portbury, Nr. Bristol. |
| Gibbs, J. R. ... ... ... ... ... ... ... ... ... | 48 Codrington Road, Bishopston, Bristol 7. |
| *Glenny, E. T., M.B., B.S. Lond. ... ... ... | 102 Pembroke Road, Clifton, Bristol 8. |
| *Gordon, R. G., M.D., B.Sc. Edin. ... ... ... | 9 The Circus, Bath. |
| *Gorham, A. P., M.B., Ch.B. Brist. ... ... ... | 53 Westbury Road, Westbury-on-Trym. |
| *Gornall, W. A., M.D., Brist. ... ... ... ... | Orchard House, Nailsea, Somerset. |
| *Gray, J. D. A., M.B., Ch.B. Edin., B.Sc. ... | 212 Redland Road, Bristol 6. |
| *Green, S. B., M.B. Lond. ... ... ... ... ... | The Black Wicket, Westbury-on-Trym. |
| *Greenlaw, Madge E., M.B., Ch.B. Brist. ... | 194 Redland Road, Bristol 6. |
| Griffiths, Cornelius A. ... ... ... ... ... ... | 35 Newport Road, Cardiff. |
| *Groves, E. W. Hey, M.D., M.S. Lond., D.Sc.... | 25 Victoria Square, Clifton, Bristol 8. |
| *Hall, E. George, M.B. Lond. ... ... ... ... | 42 Apsley Road, Clifton, Bristol 8. |
| *Hall, I. Walker, M.D., Ch.B. Vict. ... ... | Shortwood Lodge, Pucklechurch, Glos. |
| *Ham, C. I., M.B., Ch.B. Brist. ... ... ... ... | 16 Elm Lane, Redland, Bristol 6. |
| *Harris, H. Elwin, B.A., M.B. Cantab. ... ... | 13 Lansdown Place, Clifton, Bristol 8. |
| *Harris, H. E., M.A., M.B., B.Ch. Cantab. ... | 13 Lansdown Place, Clifton, Bristol 8. |
| Harrison, C. C. ... ... ... ... ... ... ... ... | 44 High Street, Keynsham. |
| *Hartley, G., M.B., B.S. Lond. ... ... ... ... | 2 Harley Place, Clifton, Bristol 8. |
| *Heales, J. N. ... ... ... ... ... ... ... ... | Royal Infirmary, Bristol. |
| *Hector, F., M.D. Lond. ... ... ... ... ... | 11 Clifton Hill, Clifton, Bristol 8. |
| *Hemphill, R., M.B., B.Ch. ... ... ... ... | Fishponds Mental Hospital, Bristol. |
| *Henderson, James, M.B., Ch.B. Aberd. ... ... | The City Sanatorium, Yardley Road, Birmingham. |
| *Herapath, C. E. K., *M.C.*, M.D. Lond. ... ... | Ormlie, Clifton, Bristol 8. |
| *Heron, Gordon, M.B., Ch B. Brist. ... ... | 34 Gloucester Road, Bristol 7. |
| *Heron, Gordon, Mrs., M.B., Ch.B. Brist. ... | 34 Gloucester Road, Bristol 7. |
| *Hooker, J. A., M.B., Ch.B. Brist. ... ... ... | 16 St. Edyth's Road, Sea Mills 9. |
| *Hosken, J. G. F. ... ... ... ... ... ... ... ... | Uley, Glos. |
| Hospital, Bristol General (*Secretary*, T. W. Gregg). | |
| *Houghton, Lydia M., M.B., B.S. Lond. ... ... | 74 Church Road, Redfield, Bristol 5. |

*Hughes, F. W. Terrell, M.B., Ch.B. Brist. ... Chew Magna, Somerset.
*Hughes, Marguerite G., M.B., Ch.B. Bris. ... Tower House, Whiteladies Road, Bristol.
*Hunter, F. S. ... ... ... ... ... ... ... ... c/o Messrs. J. Wright & Sons Ltd., Publishers, Bristol 1.
*Hyatt, Annie W., M.B., B.S. Lond. ... ... 2 Waterloo Road, Shepton Mallet, Som.

*Iles, A. E., *O.B.E.*, M.B., B.S. Lond. ... ... 17 Victoria Square, Clifton, Bristol 8.
Infirmary, Bristol Royal (*Secretary*, E. C. Smith).
Ingle, C. Durban ... ... ... ... ... ... ... Somerton, Somerset.

*Jackman, W. A., M.B., Ch.B. Brist.... ... ... 15 Mortimer Road, Clifton. Bristol 8.
James, April D., M.B., Ch.B. Brist. ... ... 8 Johnstone Street, Bath.
*James, J. A., M.D. Lond. ... ... ... ... ... 5 Rodney Place, Clifton, Bristol 8.
*James, J. A., Senr. ... ... ... ... ... ... 43 Nevil Road, Bishopston, Bristol.
*Jenkins, F. G., M.B., Ch.B. Brist. ... ... ... 51 Redcliff Hill, Bristol 1.
*Jenkins, R. D. ... ... ... ... ... ... ... ... The Copper Beeches, Almondsbury, nr. Bristol.
*Johnson, R. G., M.B. Lond. ... ... ... ... 119 Redland Road, Bristol 6.
*Joll, C. A., M.D., M.S. Lond. ... ... ... ... 64 Harley Street, London, W.1.
*Jordan, L. R., M.B., Ch.B. Brist. ... ... ... National Temperance Hospital, Hampstead Road, N.W.1.
*Joscelyne, P. C., M.B., Ch.B. Brist.... ... ... 70 Longmead Avenue, Bishopston, Bristol 7.

*Kemm, N. F., M.B., B.S. Lond. ... ... ... ... 200 Redland Road, Durdham Park, Bristol 6.
King, E. F. ... ... ... ... ... ... ... ... ... 2 Upper Wimpole Street, London, W.1.
*Kingston, S. H., M.B., Ch.B. Brist. ... ... 3 Whiteladies Road, Clifton, Bristol 8.
*Kyle, H. G., M.A., M.D., B.Ch. Oxon. ... ... 31 Westbury Road, Bristol.

*Lace, F. ... ... ... ... ... ... ... ... ... ... 5 Gay Street, Bath.
Lalonde, T. P. ... ... ... ... ... ... ... ... Wykeham House, Romsey, Hants.
*Langley, G. F., M.B., Ch.B. Brist. ... ... ... The Manse, Trentham Road, Lougton. Stoke-on-Trent.
*Lees, E. Leonard, M.D., C.M. Edin. ... ... 22 Richmond Hill, Clifton, Bristol 8.
*Legat, R. Eddowes, M.B., C.M. Edin. ... ... Murray House, Staple Hill, Bristol.
Leigh, W. W. ... ... ... ... ... ... ... ... Llansamnor House, Cowbridge, Glam.
*Levis, J. S., *M.C.*, M.B., B.Ch., N.U.I. ... ... 20 Gay Street, Bath.
*Linton, Marion S., M.B. Lond. ... ... ... 21 Oakfield Road, Clifton, Bristol 8.
*Lister, A. E. J., M.B., B.S. Lond. ... ... ... 86 Pembroke Road, Clifton, Bristol 8.
*Lister, L. Margaret ... ... ... ... ... ... 12 All Saints Road, Clifton, Bristol 8.
*Logan, H. B. ... ... ... ... ... ... ... ... ... Elmwood, Aberdeen Road, Cotham, Bristol 6.
*Lucas, J. E. ... ... ... ... ... ... ... ... ... 48 Coronation Road, Bristol 3.
*Lucas, J. J. S., M.D. Lond. ... ... ... ... 186 Wells Road, Totterdown, Bristol 4.
*Lucas, J. V. ... ... ... ... ... ... ... ... ... 186 Wells Road, Knowle, Bristol 4.
*Lucas, R. P., M.B., Ch.B. Brist. ... ... ... 15 Edgecumbe Road, Redland, Bristol 6
*Lysaght, A. C. ... ... ... ... ... ... ... ... 8 Clifton Hill, Clifton, Bristol 8.

*McCrea, Phillip, M.D., B.Ch. Dub. ... ... ... 56 Kellaway Avenue, Bristol 6.
*Macfie, J. D., M.B., Ch.B. Glasg. ... ... ... Winsley Sanatorium, near Bath.
*McKenzie, W. G., *M.C.* ... ... ... ... ... ... Ministry of Health Regional Med. Office, 3 Victoria Road, Darlington, Co. Durham.
*MacLachlan, A. M., M.B., Ch.B., Glasg. ... 65 Redcliff Hill, Bristol 1.
*McLannahan, J. G. ... ... ... ... ... ... The Mount, Stonehouse.
Maclean, Sir Ewen J., M.D., C.M. Edin. ... 12 Park Place, Cardiff.
*MacLeod, Donald, M.B., C.M. Edin. ... ... 84 Redland Road, Redland, Bristol 6.
*Macleod, G., M.A.Glas., M.D., M.B. ... ... Wargrave, Clevedon, Som.
*MacWatters, J. C., *M.B.E.* ... ... ... ... Almondsbury, near Bristol.
*Marshall, A. H., M.B., Ch.B. Bristol. ... ... Odelos, Canford Lane, Bristol.
Marshall, A. L., M.B., B.A. Cantab. ... ... Laxfield, Woodbridge, Suffolk.
*Martin, P. S. ... ... .. ... ... ... ... ... Victoria Quadrant, Weston-super-Mare.
*Maxwell, H. B. ... ... ... ... ... ... ... ... "Glencairn," Wrington, Som.
*Mayes, F. J. A. ... ... ... ... ... ... ... ... 23 Pembroke Road, Clifton, Bristol 8.
*Meadows, G. ... ... ... ... ... ... ... ... ... W. D. & H. O. Wills, No. 1 Factory, East Street, Bedminster, Bristol 3.

| | |
|---|---|
| MIALL, C. L'O. ... ... ... ... ... ... ... | Elm Hayes, Paulton, near Bristol. |
| *MILLING, T., M.B., Ch.B. Belf. .. ... ... ... | 1 Vicarage Road, Southville, Bristol 3. |
| *MILNER, A., M.B., B.Ch. Dub. ... ... ... | 61 Cotham Brow, Bristol 6. |
| *MOORE, C. A., M.B., M.S. Lond. ... ... ... | 56 St. Paul's Road, Clifton, Bristol 8. |
| *MOORE, L. A., M.B., Ch.B. ... ... ... ... ... | Hillsborough, Cotham Park, Bristol 6. |
| *MOORE, R. M., M.A., M.B., Ch.B. Cantab. ... | Mundens, Malmesbury, Wilts. |
| *MORGANS, C. C., M.B., Ch.B. ... ... ... ... | 17 First Avenue, St. Anne's Park, Bristol 4. |
| *MORRIS, L. N., M.B., Ch.B. Brist. ... ... ... | 24 All Hallows Rd., Upper Easton, Bristol 5. |
| *MULLINS, G. E. ... ... ... ... ... ... ... | 2 New Street, Wells, Som. |
| *MUNDY, G. S., M.B., B.Ch. Brist. ... ... ... | Homewood, Coombe Dingle, Bristol. |
| *MYLES, G. T. ... ... ... ... ... ... ... ... | 7 Apsley Road, Clifton, Bristol 8. |
| *MYLES, Q. St. L., M.A., B.M., B.Ch. Oxon. | 7 Apsley Road, Clifton, Bristol 8. |
| *NEWLANDS, H. J. ... ... ... ... ... ... ... | Moray House, Fishponds, Bristol. |
| *NICHOLSON-LAILEY, J. R., F.R.C.S. Eng. ... ... | 8 Mount Terrace, Taunton. |
| *NIXON, J. A., *C.M.G.*, M.D. Cantab. ... ... | 7 Lansdown Place, Clifton, Bristol 8. |
| *NOBLE, J. INNES, M.B., Ch.B. Liverp. ... ... | 102 Pembroke Road, Clifton, Bristol 8. |
| *NOTT, WINIFRED, M.B., Ch.B. Brist. ... ... ... | Fassiefern, 8 Rylestone Grove, Stoke Bishop. |
| *O'DONOHOE, MONICA A., M.B., Ch.B. ... ... | 19 Westfield, Park, Redland, Bristol 6. |
| *ORMEROD, G. L., M.A., M.B., B.Ch. Cantab. ... | 2 Henleaze Road, Westbury-on-Trym, Bristol. |
| *ORR-EWING, H. J., *M.C.*, M.D. Lond. ... ... | Beaufort Buildings, Clifton, Bristol 8. |
| *PARKER, GEORGE, M.A., M.D. Cantab. ... ... | 9 Pembroke Road, Clifton, Bristol 8. |
| *PARKES, J. A., M.B., Ch.B. Liverp. ... ... ... | 8 South Road, Redland, Bristol 6. |
| PARKINSON, Lt.-Col. G. S., *D.S.O.*, R.A.M.C. ... | R.A.M.C. Mess, Grosvenor Place, London, S.W.1. |
| *PARRY, R. H., M.B., B.S. Lond. ... ... ... | Public Health Offices, 40 Prince Street, Bristol 1. |
| PARSONS, SIR J. H., M.B., B.S., B.Sc. Lond. ... | 54 Queen Anne Street, London, W. |
| *PAUL, R. G. ... ... ... ... ... ... ... ... ... | The Royal Infirmary, Bristol 1. |
| *PERRY, BRUCE, M.D., Ch.B. Brist. ... ... ... | 4 Richmond Park Road, Clifton, Bristol 8. |
| *PHILLIPS, P., M.B., Ch.B. Brist. ... ... ... ... | Southmead Hospital, Bristol. |
| *PINEO, E. D. ... ... ... ... ... ... ... ... | Richmond House, Langford, Som. |
| *POLLARD, J., M.B., B.Ch., B.A.O., N.U.I. ... | 88 Coronation Road, Bristol 3. |
| PORTER, C. P. ... ... ... ... ... ... ... ... | 28 Mill Street, Kidderminster. |
| *POTTER, MABEL F., M.B., Ch.B. ... ... ... | 23 Pembroke Road, Clifton, Bristol 8. |
| *POWELL, H. F., M.D. Brux. ... ... ... ... | Ellenborough Park, Weston-super-Mare. |
| *POWELL, J. J., M.D. Lond. ... ... ... ... | 2 Gloucester Road, Bishopston, Bristol 7. |
| *PRICE, A., M.B., Ch.B. Brist. ... ... ... ... | 16 Cavendish Road, Henleaze, Bristol. |
| *PRICE, C. H. G., M.B., Ch.B. Brist. ... ... ... | 152 Coronation Road, Bristol 3. |
| *PRICE, N. L., M.B., Ch.B. Brist. ... ... ... | 37 Henleaze Avenue, Bristol. |
| *PRIDIE, K. H., M.B., B.S. Lond. ... ... ... | 40 Apsley Road, Clifton, Bristol 8. |
| *PRINGLE, Miss J. L., M.B., Ch.B. (Edin.) ... | 115 Lower Oldfield Park, Bath. |
| *PROWSE, D. C., M.B., Ch.B. Brist. ... ... ... | Wigmore House, Thornbury, nr. Bristol. |
| *PYKE, H. D., M.B., Ch.B.Brist.... ... ... ... | 88 Redland Road, Bristol 6. |
| *RANKIN, P. C., M.B., Ch.B.Glasg. .. .. .. | Lawrence Hill House, Bristol. |
| *RAYNER, D. C., Ch.M. Brist. ... ... ... ... | 9 Lansdown Place, Clifton, Bristol 8. |
| *REDMAN, ETHEL, M.B., Ch.B. ... ... ... ... | 55 Bridgwater Road, Bedminster Down, Bristol 3. |
| *ROBERTSON, D., M.B., Ch.B. Edin. ... ... ... | 205 Wells Road, Knowle, Bristol 4. |
| *ROBERTSON, L. G., M.B., Ch.B. ... ... ... | 85 Ashley Road, Bristol 6. |
| *ROGERS, HERBERT, M.D., Brist. ... ... ... | 11 Clifton Hill, Bristol 8. |
| *ROLFE, R., B.A., M.B., B.C. Cantab. ... ... | Park House, St. Andrew's Road, Avonmouth. |
| *ROWLEY, A. T. ... ... ... ... ... ... ... | 13 Walliscote Road, Weston-super-Mare. |
| *RUDOLF, G. R. A. de M. ... ... ... ... ... | Brentry Colony, Westbury-on-Trym, Bristol. |
| SAVAGE, W. G., M.D., B.Sc. Lond. ... ... ... | "Bourn," 7 Trewartha Park, Weston-super-Mare. |
| *SAXBY, IDA, D.Sc., Lond. ... ... ... ... ... | 3 Westbury Park, Bristol 6. |
| *SCARFF, G., M.B., Ch.B. Edin. ... ... ... ... | 83 Pembroke Road, Clifton, Bristol 8. |

| | |
|---|---|
| *SHAW, J. E., M.B., C.M. Edin. | 23 Caledonia Place, Clifton, Bristol 8. |
| *SHEPHERD, H. L., M.B., Ch.M. Brist. | 79 Pembroke Road, Clifton, Bristol 8. |
| *SHORT, A. R., B.Sc., M.D., B.S. Lond. | 69 Pembroke Road, Clifton, Bristol 8. |
| *SMITH, G. COCKBURN, M.D. Brux. | 12 South Road, Newton Abbot. |
| *SMITH, T. WILSON, M.D. Lond. | 26 The Circus, Bath. |
| *SMYTHE, H. J. D., *M.C.*, M.D., M.S. Lond. | 15 Eaton Crescent, Clifton, Bristol 8. |
| *SOMERS, MARY A. E. | 2 Ashcombe Road, Weston-super-Mare. |
| *SPOOR, A., M.B., B.Ch. Cantab. | The Hydro, Bristol 1. |
| *STANILAND, M. F. | 255 Stapleton Road, Bristol 5. |
| *STATHAM, R. S. S., *O.B.E.*, M.D., M.Ch. Brist. | Cheddar, Som. |
| *STEVEN, G. D., M.B., Ch.B.Edin., D.P.H. | 11 North Parade, Bath. |
| *STONE, D. M., M.D., B.S. Lond. | 2 Harley Place, Clifton, Bristol 8. |
| *STROVER, H. W. M., *O.B.E.*, M.B., Ch.B. Aberd. | 12 Gloucester Road, Bishopston, Bristol 7. |
| *STRUTHERS, A. J., M.B., Ch.B. Glasg. | 100 Church Road, Redfield, Bristol 5. |
| *SUTTON, G. E. F., M.D. Lond. | 1 Pembroke Road, Clifton, Bristol 8. |
| *SYMES, J. O., M.D. Lond. | 6 Pembroke Vale, Clifton, Bristol 8. |
| *TASKER, D. G. C., M.B., M.S. Lond. | 9 College Fields, Clifton, Bristol 8. |
| *TAYLOR, A. L., M.D. Lond. | The General Hospital, Bristol 1. |
| TAYLOR, A. L. | The Royal Infirmary, Bristol 1. |
| *TAYLOR, CONSTANCE L., M.B., Ch.B.Edin. | Queen Charlotte's Hospital, Marylebone Road, N.W. 1. |
| *TAYLOR, H. N., M.A. Cantab., M.D. Dub. | Hillside, Ebbw Vale. |
| *TEMPLE, G. H., M.B., C.M. Edin. | Ailanthus, Weston-super-Mare. |
| THIN, JAMES | 54 & 55 South Bridge, Edinburgh. |
| *THOMAS, J. D., M.B. Cantab. | Dan-y-Graig, Cheyne Road, Stoke Bishop Bristol 9. |
| *THOMPSON, J., M.B., Ch.B. Edin. | Eastbourne House, Devizes. |
| *TODD, A. T., *O.B.E.*, M.B., Ch.B. Brist. | Royal Park House, Clifton, Bristol 8. |
| TRIPP, DOROTHY M. H. | Methodist Missionary Hospital, Medak, H.E.H. Nizam's Dominions, India. |
| *TRIST, J. R. R., *M.C.* | 120 Henleaze Road, Henleaze. |
| *TRYON, VICTORIA S., M.B., Ch.B. Brist. | 3 Harley Place, Clifton, Bristol 8. |
| *WALBANK, A., M.B., Ch.B. Leeds | 30 Whiteladies Road, Clifton, Bristol 8. |
| *WALKER, CYRIL H., B.A., M.B. Cantab. | Harewood, Clapton-in-Gordano, Som. |
| *WALTERS, C. F. | 5 Mortimer Road, Clifton, Bristol 8. |
| *WANSBROUGH, T. B., M.B., Ch.B. Brist. | 8 Mortimer Road, Clifton, Bristol 8. |
| WAKE, S. C. | Brantwood, Bideford, N. Devon. |
| *WARD, H. W., F.R.C.S. Edin. | Chipping Manor, Wotton-under-Edge. |
| *WARREN, R., M.A., M.D. Oxon. | Ellenborough Hall, Weston-super-Mare. |
| *WATERHOUSE, R., M.D. Lond. | 3 The Circus, Bath. |
| *WATSON-WILLIAMS, E., *M.C.*, B.A., M.B., Ch.M. Cantab. | 12 Victoria Square, Clifton, Bristol 8. |
| WATTS J. B. V. | 6 Queen Street, Cambridge, C.P., South Africa. |
| *WEDDELL, C. W. | Boydville, 46 Miller St., West Melbourne, Australia. |
| WESTON, B. A., M.B. (see Astley-Weston). | |
| *WHELTON, A., B.A., M.B., Ch.B. Cork | Innisfree, Western Road, Cork. |
| *WHITE, E. B. C. | Mental Hospital, Fishponds, Bristol. |
| WHITEHOUSE, H. BECKWITH, M.S. Lond. | 62 Hagley Road, Edgbaston, Birmingham. |
| *WIGODER, SYLVIA BEATRICE, M.A., M.D., Ph.D. | Radium Centre Royal Infirmary, Bristol 1. |
| *WILBOND, R. G. | 92 Chesterfield Road, St.Andrew's, Bristol. |
| WILLCOX, R. LEWIS | 97 London Road, Salisbury. |
| *WILLIAMS, A. R., M.B., Ch.B. Brist. | West Lodge, Frome, Som. |
| *WILLIAMS, I., M.B., Ch.B. Brist. | "Ardrem," Hill View, Henleaze, Bristol. |
| WILLIAMS, S. R., M.B. Lond. | Buckfastleigh, Devon. |
| *WILLS, W. K., *O.B.E.*, M.B., B.C. Cantab. | 1 Victoria Square, Clifton, Bristol 8. |
| *WOOD, DUNCAN | 105 Pembroke Road, Clifton, Bristol 8. |
| *WOOD, W. V., *M.C.*, B.A. Cantab. | Henley Lodge, Yatton, Som. |
| *WRIGHT, A. J. M.B., B.S. Lond. | 2 Clifton Park, Clifton, Bristol 8. |
| *WRIGHT, WINIFRED M., M.B., B.S. Lond. | 2 Waterloo Road, Shepton Mallet, Som. |
| *ZEALAND, L., M.D. Man. | Brecon Lodge, Westbury-on-Trym, Bristol. |

